# Brewers' Rice: A By-Product from Rice Processing Provides Natural Hepatorenal Protection in Azoxymethane-Induced Oxidative Stress in Rats

**DOI:** 10.1155/2015/539798

**Published:** 2015-07-14

**Authors:** Bee Ling Tan, Mohd Esa Norhaizan, Ithnin Hairuszah, Hamzah Hazilawati, Karim Roselina

**Affiliations:** ^1^Department of Nutrition and Dietetics, Faculty of Medicine and Health Sciences, Universiti Putra Malaysia, 43400 Serdang, Selangor, Malaysia; ^2^Research Centre of Excellent, Nutrition and Non-Communicable Diseases (NNCD), Faculty of Medicine and Health Sciences, Universiti Putra Malaysia, 43400 Serdang, Selangor, Malaysia; ^3^Laboratory of Molecular Biomedicine, Institute of Bioscience, Universiti Putra Malaysia, 43400 Serdang, Selangor, Malaysia; ^4^Department of Pathology, Faculty of Medicine and Health Sciences, Universiti Putra Malaysia, 43400 Serdang, Selangor, Malaysia; ^5^Department of Veterinary Pathology and Microbiology, Faculty of Veterinary Medicine, Universiti Putra Malaysia, 43400 Serdang, Selangor, Malaysia; ^6^Department of Food Technology, Faculty of Food Science and Technology, Universiti Putra Malaysia, 43400 Serdang, Selangor, Malaysia

## Abstract

Brewers' rice, which is known locally as *temukut*, is a mixture of broken rice, rice bran, and rice germ. Our present study was designed to identify the effect of brewers' rice on the attenuation of liver and kidney damage induced by azoxymethane (AOM). Alanine transaminase (ALT), alkaline phosphatase (ALP), aspartate transaminase (AST), creatinine, and urea were evaluated to understand potential hepatoprotective effects and the ability of brewers' rice to attenuate kidney pathology induced by AOM treatment. Liver and kidney tissues were evaluated by hematoxylin and eosin (H&E) staining. Overall analyses revealed that brewers' rice improved the levels of serum markers in a manner associated with better histopathological outcomes, which indicated that brewers' rice could enhance recovery from hepatocyte and kidney damage. Taken together, these results suggest that brewers' rice could be used in future applications to combat liver and kidney disease.

## 1. Introduction

The liver is a critical organ that plays an essential role in metabolism, storage, and excretion of metabolites. Several hepatotoxins contribute to liver damage, including ethanol, paracetamol, and carbon tetrachloride (CCl_4_) [1, 2]. Azoxymethane (AOM) is an active metabolite of the colon-specific carcinogen 1,2-dimethylhydrazine (DMH) that effectively induces colon tumors in susceptible rodents. AOM also causes hepatotoxicity in various experimental animal studies [3] and is responsible for most of the damage in the liver [4] and kidney [5] during the induction of colon cancer.

Oxidative stress is a primary factor in the development of pathological lesions during liver disease [6] and can cause excessive damage to hepatocytes by lipid peroxidation and protein alkylation [7]. Upon hepatotoxin induction, Kupffer cells release proinflammatory mediators, such as nitric oxide (NO) and interferon-gamma (IFN-*γ*), which subsequently result in the accumulation of reactive oxygen species (ROS). ROS have been reported to cause lipid peroxidation and membrane degradation, which contributes to inflammation and liver damage [1].

Polyphenols and flavonoids possess strong antioxidant activities and are demonstrably protective against liver injury in rats [2, 8]. Brewers' rice, known locally as* temukut*, consists of broken rice, rice bran, and rice germ and accounts for nearly 1% of total rice. It is typically used as animal feed and brewing material [9]. The production of brewers' rice during rice milling is described in previous study by Esa et al. [10]. Recent study has found that water extract of brewers' rice (WBR) induced apoptosis in a human colorectal cancer (HT-29) cell line [11]. These antiproliferative effects of WBR may potentially be due to the presence of bioactive antioxidant polyphenolic compounds [12]. We also determined brewers' rice to be an effective dietary agent for the reduction of colon tumor incidence and multiplicity formation in AOM-treated rats [13]. To date, no study has assessed the beneficial effects of brewers' rice on liver and kidney injuries. Thus, the purpose of this study was to investigate whether brewers' rice protects against liver and kidney injuries during colon carcinogenesis induction with AOM in male Sprague-Dawley rats.

## 2. Materials and Methods

### 2.1. Chemicals and Reagents

AOM, phosphate-buffered saline (PBS), and 10% (v/v) neutral buffered formalin were bought from Sigma (St. Louis, MO, USA). All other chemicals and reagents used were of analytical grade and from Sigma-Aldrich (St. Louis, MO, USA).

### 2.2. Sample Preparation

Freshly milled brewers' rice samples (rice variety MR 219) were supplied by the BERNAS Milling Plant (Seri Tiram Jaya, Selangor, Malaysia). Stabilization of brewers' rice was performed as previously reported by Tan et al. [12]. Brewers' rice consists of broken rice (95.16 ± 4.62%), rice bran (3.60 ± 0.39%), and rice germ (1.11 ± 0.07%).

### 2.3. Animals and Diets

**Figure 1:**
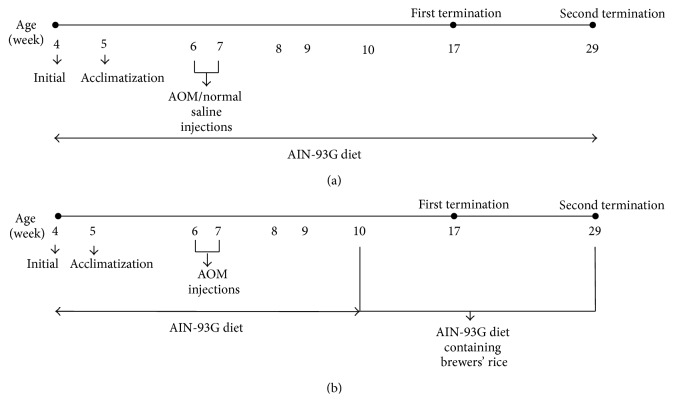
Experimental design of the animal study. (a) Time line of experiment for Group 1 and Group 2. (b) Time line of experiment for Groups 3, 4, and 5. First termination = after 8 weeks of treatment with brewers' rice; second termination = after twenty weeks of treatment with brewers' rice; AIN = American Institute of Nutrition; AOM = azoxymethane.

A total of 60 four-week-old male Sprague-Dawley rats (*Rattus norvegicus*) weighing approximately 90–100 grams were housed in plastic cages (two rats per cage) with wood-chip bedding in a fully ventilated room with 12-hour light/dark cycles at approximately 25–27°C and 50 ± 10% relative humidity. This study was conducted in compliance with the guidelines approved by the Animal Care and Use Committee (ACUC) of the Faculty of Medicine and Health Sciences, Universiti Putra Malaysia (UPM) Serdang, Selangor (Reference number UPM/FPSK/PADS/BR-UUH/00461). The rats were acclimatized for seven days and received with the American Institute of Nutrition (AIN)-93G diet and tap water* ad libitum*. The rats were randomly divided into five groups (*n* = 12 rats for each group) that included the following: (G1), normal; (G2), AOM only; (G3), AOM + 10% (w/w) brewers' rice; (G4), AOM + 20% (w/w) brewers' rice; and (G5), AOM + 40% (w/w) brewers' rice. At six weeks of age, the animals in groups G2 to G5 received intraperitoneal injections of AOM (15 mg/kg body weight) once weekly over a 2-week period. The rats in the normal group received an equal volume of normal saline that served as a vehicle control. The control groups (G1 and G2) fed an AIN-93G diet throughout the experiment. Groups G3, G4, and G5 fed an AIN-93G diet containing 10%, 20%, and 40% (w/w) brewers' rice, respectively. The components of the AIN-93G diet were modified following the nutrient composition of brewers' rice as described by Tan et al. [13] (Table 1). Experimental design of the animal study is demonstrated in Figure 1.

### 2.4. Carcinogenesis Induction

The carcinogen azoxymethane (AOM) was diluted in 0.9% (v/v) saline. The rats received intraperitoneal injections once weekly (15 mg/kg body weight) over a 2-week period [14].

### 2.5. Serum Biochemistry

After twenty weeks of treatment, six rats from each group were sacrificed after anesthetization with diethyl ether. Blood samples from all groups were collected in containers without anticoagulant, thus allowing clot formation. The blood was centrifuged at 1,200 ×g for 10 minutes. The serum was stored at −80°C until further analysis. The activities of blood serum marker enzymes, such as alanine transaminase (ALT), alkaline phosphatase (ALP), aspartate transaminase (AST), creatinine, and urea, were measured using a Roche kit (Penzberg, Germany) and analyzed spectrophotometrically using the Hitachi Analytical Instrument (Roche Diagnostic GmbH, Mannheim, Germany).

### 2.6. Organ Collection for Histological Studies

Lobes from the liver and the kidney tissues of each animal were collected for histologic studies after eight and twenty weeks of treatment with brewers' rice. The organ tissues were flushed with PBS and fixed in 10% (v/v) neutral buffered formalin for further analyses. The tissues were trimmed, embedded in paraffin, sectioned at 4–6-*µ*m thickness using a Leica rotation microtome, and stained with hematoxylin and eosin (H&E) for histopathological evaluation. Histopathological assessment was performed on all liver and kidney tissue samples. Liver pathology was graded using a modified method from Chiou et al. [15]. Histopathological changes in liver tissues were graded using the following scale of 0–9 according to the severity of histopathological alterations observed: 0 = preservation of normal architecture and histology of the liver; 1 = mild hydropic degeneration or fatty changes in ≤20% of cells; 2 = moderate hydropic degeneration or fatty changes in 21–50% of cells; 3 = severe hydropic degeneration or fatty changes in >50% of cells; 4 = mild inflammation (few foci of inflammatory infiltrates in ≤20% of cells); 5 = moderate inflammation (multiple foci of inflammatory infiltrates in 21–50% of cells); 6 = severe inflammation (diffuse foci of inflammatory infiltrates in >50% of cells); 7 = mild necrosis (focal necrotic foci involving one location); 8 = moderate necrosis (multiple necrotic foci involving more than one location); 9 = severe necrosis (diffuse, confluent, and bridging). Each histopathological lesion was further divided into mild, moderate, or severe categories. The score for each sample was determined by an examination of the most severe field selected from each liver section using a light microscope with a 100x magnification.

Histopathological changes in the kidney tissues were graded using a modified method from Chiou et al. [15] based on vacuolation and the degeneration of the tubules, tubular dilatation, and deposition of protein cast within the tubular lumina of the kidney, degree of inflammation, and degree of necrosis. The vacuolation and the degeneration of the tubules observed in the kidney were examined using the following scale: 0 = no changes; 1 = mild changes in ≤20% of cells; 2 = moderate changes in 21–50% of cells; 3 = severe changes in >50% of cells. Tubular dilatation and deposition of protein cast within the tubular lumina of the kidney observed in the kidney were examined using the following scale: 0 = no changes; 1 = mild changes in ≤20% of cells; 2 = moderate changes in 21–50% of cells; 3 = severe changes in >50% of cells. Degree of inflammation observed in the kidney was assessed using the following scale: 0 = no inflammation; 1 = mild inflammation (few foci of inflammatory infiltrates in ≤20% of cells); 2 = moderate inflammation (multiple foci of inflammatory infiltrates in 21–50% of cells); 3 = severe inflammation (diffuse foci of inflammatory infiltrates in >50% of cells). Degree of necrosis observed in the kidney was assessed using the following scale: 0 = no necrotic foci; 1 = mild necrosis (focal necrotic foci involving one location); 2 = moderate necrosis (multiple necrotic foci involving more than one location); 3 = severe necrosis (diffuse). The kidney lesion scores were assessed under a light microscope using a 100x magnification.

### 2.7. Statistical Analysis

The statistical data were expressed as the mean ± standard deviation (SD) and performed using one-way analysis of variance (ANOVA) with Tukey's multiple range tests. Statistical analyses were conducted using the Statistical Package for Social Science (SPSS) version 17.0. A *P* value of <0.05 was considered significant.

## 3. Results and Discussion

### 3.1. Relative Organ Weight

**Figure 2:**
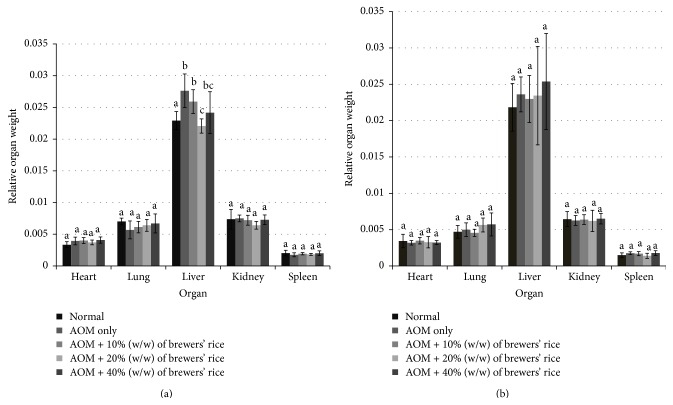
Relative organ weight of rats. (a) After eight-week treatment with brewers' rice. (b) After twenty-week treatment with brewers' rice. Value with different superscript letter indicates significant difference between groups by Tukey's test (*P* < 0.05).

Organ weights were measured for all groups and expressed as relative organ weights [16]. As shown in Figures 2(a) and 2(b), the final relative heart, lung, liver, kidney, and spleen weights were measured at the termination of the study (after eight and twenty weeks of treatment with brewers' rice). After eight weeks of treatment with brewers' rice, no significant difference was found in the relative heart, lung, kidney, and spleen weights among the normal, AOM only, or brewers' rice groups (*P* > 0.05). However, a significant increase in relative liver weight was observed in the AOM-only group and two treatment groups (10% and 40% (w/w) of brewers' rice) compared to that observed in the normal group (*P* < 0.05) (Figure 2(a)). Relative organ weight depends on the organ weight and body weight of individual rat. Even though an earlier study demonstrated that AOM causes hepatotoxicity in various experimental animal studies [3] and is responsible for most of the damage in the liver [4] during the induction of colon cancer, which could reduce the body weight of rats. However, in the present study, the increase of relative liver weight in AOM-only group and those groups fed with diets containing 10% (w/w) brewers' rice and 40% (w/w) of brewers' rice may reveal that the study duration was too short to observe the previously shown effects. The relative heart, lung, liver, kidney, and spleen weights for all brewers' rice-fed groups were nearly similar to those of the normal group and did not show any significant differences after twenty weeks of treatment with brewers' rice (*P* > 0.05) (Figure 2(b)). The absence of significant differences in relative organ weight after twenty weeks of treatment with brewers' rice implied that dietary intake had no apparent influence on the findings.

### 3.2. Effect of Brewers' Rice on Liver and Kidney Function Biomarkers

To explore whether dietary administration of brewers' rice modulated the development of liver and kidney lesions during AOM-induced colon carcinogenesis, male Sprague-Dawley rats were provided with different doses (10%, 20%, and 40% (w/w)) of brewers' rice. A dosage of 10% (w/w) brewers' rice was suggested by Boateng et al. [17] in a study regarding rice bran and germ in which this concentration was reported to inhibit colon tumor incidence. Additionally, higher brewers' rice concentrations (20% and 40% (w/w) brewers' rice) were selected in this study to examine the dose-dependent effect of brewers' rice as a dietary agent. Our previous study showed that dietary administration up to 40% (w/w) of brewers' rice is well tolerated and did not inhibit the growth of rats [13].

Liver damage can be categorized as direct hepatocyte destruction or bile flow impairment [18]. Cytoplasmic enzymes in the hepatocytes may leak into the blood during the early stages of liver damage when membrane permeability increases [19]. The most common biomarkers used to evaluate liver function are ALT, ALP, and AST [20, 21]. Thus, the effects on serum ALT, ALP, and AST in AOM-induced liver toxicity after twenty weeks of treatment with brewers' rice were evaluated.

The effects of twenty weeks of dietary administration of brewers' rice on liver and kidney serum biomarkers during AOM-induced toxicity were evaluated, and the results are summarized in Table 2. Serum biomarker evaluation revealed that the highest ALT levels were observed in the AOM-only group compared to the groups treated with brewers' rice. The serum ALT level was successfully reduced in rats fed with brewers' rice. The moderate reduction in ALT levels may be due to a low dose (10% (w/w)) of brewers' rice fed to the rats. However, the ALT level did not significantly differ between the AOM-only group and those groups fed with diets containing 10% (w/w) brewers' rice, 20% (w/w) brewers' rice, or 40% (w/w) brewers' rice (*P* > 0.05). This finding indicated that serum ALT was decreased in a dose-dependent manner after feeding with brewers' rice during AOM-induced liver toxicity.

As shown in Table 2, in the AOM-only group, the mean ALP level was 164.00 U/L. Analysis of serum biomarkers revealed that brewers' rice administration decreased ALP levels, with a maximum reduction observed in the rats fed 40% (w/w) brewers' rice. ALP levels observed in the rats fed 40% (w/w) brewers' rice were nearly similar to those of the normal group. However, no significant difference was found in the ALP levels between the AOM-only group and those from the groups fed 10% (w/w) brewers' rice or 20% (w/w) brewers' rice (*P* > 0.05). Moreover, a reduction in AST levels was observed in the groups treated with brewers' rice. The AST level in the AOM-only group was higher than those observed in the brewers' rice-fed groups, but this difference was not significant (*P* > 0.05) (Table 2).

Serum urea and creatinine were measured as indicators of kidney function [22]. As shown in Table 2, the highest creatinine level was observed in the AOM-only group. After twenty weeks of brewers' rice administration, the creatinine level was reduced in the groups treated with brewers' rice. No significant difference was observed in the creatinine levels among the AOM-only group and the brewers' rice groups (10%, 20%, 40% (w/w) of brewers' rice) (*P* > 0.05). A similar trend (no significant difference) was also observed in urea levels among the AOM-only group and the 10%, 20%, and 40% (w/w) brewers' rice groups (*P* > 0.05). This finding indicated that the highest dose of brewers' rice (40% (w/w) brewers' rice) was able to maintain the serum ALT, ALP, creatinine, and urea levels closest to the normal. Taken together, the data presented in this study suggest that brewers' rice has the potential to reduce liver serum biomarkers including ALT, ALP, and AST.

### 3.3. Histopathological Evaluation of the Liver after Eight and Twenty Weeks of Treatment

The liver is the most common site of colon cancer metastases [23]. Three high doses (15–25 mg/kg body weight) of AOM promote the growth of tumors in the gastrointestinal tract, auditory sebaceous glands, liver, kidney, and preputial gland [24]. AOM is mainly metabolized by the CYP2E1 isoform of cytochrome P450 [25]. The first step in AOM metabolism involves the hydroxylation of the methyl group of AOM to form methylazoxymethanol (MAM). MAM breaks down into formaldehyde and a highly reactive alkylating species, usually methyldiazonium. This chemical causes the alkylation of guanine to O6-methylguanine (O6-MEG) and O4-methylthymine [26]. A study reported that AOM has been used in animal studies to evaluate the preventative potential for treatments following AOM-induced carcinogenesis [27]. Serum enzymes, such as ALT, ALP, and AST, are not specific, but increases in the activities of these enzymes are associated with active destruction of the liver [28]. Because biochemical measurements alone are inconclusive, histopathological evaluations were also required to prove the protective efficacy of brewers' rice on the liver and kidney lesions.

Hepatocellular damage scores observed throughout the experiment are summarized in Table 3. The histopathological changes in rat tissues were compared among all groups throughout the study. Destructive changes in liver sections were most evident in rats only injected with AOM and not administered brewers' rice at any dose, as they exhibited the highest histopathology scores upon H&E staining. After eight-week administration of brewers' rice, our data revealed that the livers of rats in the brewers' rice treatment groups demonstrated notable recovery from AOM-induced liver damage when compared to the livers of the AOM-only group, but this difference was not significant (*P* > 0.05).

Consistent with the effects observed after eight weeks of treatment, liver tissues from the normal group also exhibited mild to moderate fatty changes within the hepatocytes after the twenty-week time points. The observed fatty changes in the hepatocytes of the normal group are associated with the dysregulation of mitochondrial *β*-oxidation of fatty acids. This subsequently resulted in the esterification of fatty acids to triglyceride in the cytoplasm, which is characterized by the presence of triglyceride droplets within the hepatocytes [29]. However, our study contradicted with that of Mohd Ali et al. [30], who found no pathological abnormalities in the livers of normal mice. After twenty weeks of treatment with brewers' rice, the liver tissues from the AOM-only group had the highest histopathology scores among all groups (Table 3). A significant reduction in liver damage was observed in the two treatment groups (20% and 40% (w/w) of brewers' rice) compared with the liver tissues of the AOM-only group (*P* < 0.05). However, no significant difference was found between the AOM-only liver samples and those from the 10% (w/w) brewers' rice treatment group (*P* > 0.05) (Table 3).

**Figure 3:**
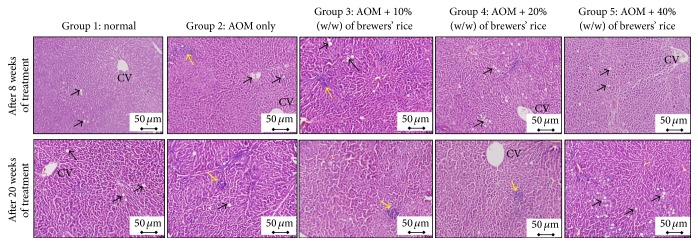
Histological characterization of liver tissue of rats from different experimental groups (magnification 200x). After eight weeks of treatment, normal group which received normal saline shows a mild fatty change within the hepatocytes (black arrow); AOM-only group shows a moderate inflammation and small localized inflammatory infiltrates (yellow arrow) surrounded by mild to moderate fatty changes (black arrow); dietary administration of 10% (w/w) brewers' rice in the AOM-treated rats shows moderate inflammation (yellow arrow) and mild fatty changes (black arrow); dietary administration of 20% (w/w) brewers' rice in the AOM-treated rats shows moderate fatty changes (black arrow); and dietary administration of 40% (w/w) brewers' rice in the AOM-treated rats shows mild fatty changes (black arrow). After twenty weeks of treatment, normal group shows a mild fatty change within the hepatocytes (black arrow); AOM-only group shows a moderate inflammation and small localized inflammatory infiltrates (yellow arrow) surrounded by mild fatty changes (black arrow); dietary administration of 10% (w/w) brewers' rice in the AOM-treated rats shows moderate inflammation (yellow arrow); dietary administration of 20% (w/w) brewers' rice in the AOM-treated rats shows mild inflammation (yellow arrow); and dietary administration of 40% (w/w) brewers' rice in the AOM-treated rats shows moderate fatty change (black arrow). Centrilobular vein (CV).

Histopathological evidence demonstrated that in the liver section of the rats from the AOM-only group, in which carcinogenesis was induced but no brewers' rice treatment was administered, moderate inflammation and small localized inflammatory infiltrates could be visualized (Figure 3). Strikingly, in the liver sections from the AOM-only group, various stages of fatty change and inflammation could be observed and indicated the early phases of liver injury. This finding was consistent with a study reported by Humpage et al. [31], who demonstrated that AOM-treated mice exhibited pathological abnormalities in the liver, such as inflammation. However, the liver histology of AOM-treated rats that were also administered brewers' rice diets showed notable recovery from AOM-induced liver damage with less inflammation and fewer fatty changes when compared to the rats from the AOM-only group (Figure 3).

In the present study, a large number of fatty changes were observed in the livers of AOM-treated rats. An increasing number of vacuoles in the liver might be associated with liver toxicity, and this usually indicates fatty infiltration or steatosis [32]. Hallmarks of liver injury, such as the fatty changes and inflammation, observed in the AOM-only group, were nearly eliminated after administration with the highest dose of brewers' rice (40% (w/w)). These results were consistent with declines in ALT and ALP levels. The correlation between liver biomarkers and histological changes in liver tissues further proved that these markers can be used for early detection of acute liver damage. A reduction in biochemical and histopathological lesions in the groups treated with brewers' rice diets demonstrated the hepatoprotective properties of brewers' rice. This protective effect may be associated with several of the bioactive compounds that are present in the brewers' rice via metabolic activation and detoxification of AOM. Taken together, the data presented in this study suggest that brewers' rice may represent a promising natural dietary agent in the reduction of liver damage.

### 3.4. Histopathological Evaluation of the Kidney after Eight and Twenty Weeks of Treatment

**Figure 4:**
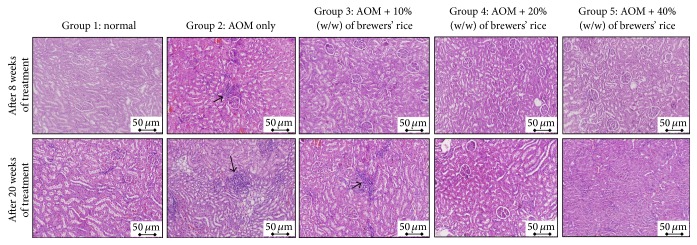
Morphological evaluations of kidney histology in different groups of rat (magnification 200x). After eight weeks of treatment, normal group which received normal saline shows a normal architecture and histology of the kidney; AOM-only group shows a mild inflammation (black arrow); dietary administration of 10%, 20%, and 40% (w/w) brewers' rice in the AOM-treated rats shows normal kidney tissue appearance. After twenty weeks of treatment, normal group which received normal saline shows a normal architecture and histology of the kidney; AOM-only group and dietary administration of 10% (w/w) brewers' rice in the AOM-treated rats show mild inflammation (black arrow); dietary administration of 20% and 40% (w/w) brewers' rice in the AOM-treated rats shows normal kidney tissue appearance.

AOM has been demonstrated to cause histopathological alterations not only in the colon and liver but also in the kidney [33]. To ascertain whether brewers' rice suppressed kidney lesions in AOM-treated rats, we further examined histological changes in the kidney samples. As shown in Table 4, kidney histopathology of the normal rats fed a control diet showed normal kidney architecture after eight weeks of treatment. Histopathological evaluation using H&E staining demonstrated that kidney inflammation was only present in the AOM-only group and not in those groups fed brewers' rice diets (Figure 4). As expected, none of the rats (normal group, AOM-only group, and brewers' rice-fed groups) developed vacuolation or the degeneration of the tubules, tubular dilatation, and deposition of protein cast within the tubular lumina of the kidney or necrosis (Table 4).

In addition to the effects observed with regard to kidney damage after eight weeks of treatment, we found that none of the rats in the normal group exhibited kidney inflammation when autopsied after twenty weeks of treatment. The kidney tissues samples obtained from the normal group appeared normal. Dietary administration of 20% and 40% (w/w) brewers' rice in the AOM-treated rats restored normal kidney tissue appearance, as no abnormalities were observed in the kidneys of rats treated with 20% and 40% (w/w) brewers' rice (Figure 4). In brewers' rice-treated rats, it was evident that the attenuation of inflammation was increased in a dose-dependent manner (Table 5). However, the extent of inflammation in the kidney did not significantly differ between the AOM-only group and those fed diets containing 10% (w/w) brewers' rice (*P* > 0.05). Consistent with the kidney lesions observed in rats after eight weeks of treatment, we found that none of the rats (normal group, AOM-only group, and brewers' rice-fed groups) developed vacuolation or the degeneration of the tubules, tubular dilatation, and deposition of protein cast within the tubular lumina of the kidney or necrosis. This finding indicated that sufficient time was required for noticeable kidney damage to be induced by AOM treatment.

In the present study, histological evaluation using H&E staining was completed to complement the data from serum biochemistry profiles. The histological changes found in the liver and kidney tissues during AOM-induced toxicity supported the biochemistry results obtained. Administration of AOM in this rat model revealed elevated liver and kidney function biomarkers, such as ALT, ALP, AST, creatinine, and urea, along with increased histopathology scores. However, possible hepatoprotective effects and attenuation of kidney damage were observed in rats treated with a diet containing brewers' rice.

A plausible justification to explain this hepatoprotection and amelioration of kidney injury may be the presence of bioactive compounds, such as phenolic antioxidants, phytic acid, vitamin E, *γ*-oryzanol [12], and dietary fiber [13], found in brewers' rice, which were reported in our earlier studies. Furthermore, we also identified that brewers' rice contained 2.30 ± 0.63 *μ*g/g *γ*-aminobutyric acid (GABA) (unpublished results). A previous study reported that GABA provides liver protection via the maintenance of intracellular polyamines during ethanol and CCl_4_-induced hepatic lesions [34, 35]. Butyric acid from the fermentation of dietary fiber plays a critical role in liver cell tumor repair [36]. In addition, *γ*-oryzanol has strong antioxidant activity [37] that has been reported to exert numerous biological effects and act as an anti-inflammatory agent [38, 39]. Antioxidants have also been found to reduce kidney injury following oxidative challenge [40]. Overall, this finding suggests that the protective effect of brewers' rice on AOM-induced liver and kidney oxidative stress may be mediated partly via the synergistic/additive effects of these bioactive constituents.

## 4. Conclusions

Our study indicated that brewers' rice has a great potential for alleviating histological injuries in AOM-induced liver and kidney pathology. Moreover, because brewers' rice is edible, thus it can be used in a variety of food products such as noodles and breakfast cereal. However, further investigations are needed to elucidate the underlying mechanisms of action that are associated with these protective effects. Taken together, these results suggest that brewers' rice could be used in future applications to combat liver and kidney disease.

## Figures and Tables

**Table 1:** Composition of experimental diets.

Ingredients (g/1000 g diet)	Group				
	G1	G2	G3	G4	G5
Brewers' rice	—	—	100.0	200.0	400.0
Corn starch	397.5	397.5	315.3	233.2	68.9
Casein	200.0	200.0	191.0	182.0	164.0
Maltodextrin	132.0	132.0	132.0	132.0	132.0
Sucrose	100.0	100.0	100.0	100.0	100.0
Soybean oil	70.0	70.0	68.1	66.1	62.2
Powdered cellulose	50.0	50.0	44.7	39.4	28.7
AIN-93G mineral mix	35.0	35.0	33.4	31.9	28.8
AIN-93G vitamin mix	10.0	10.0	10.0	10.0	10.0
L-cystine	3.0	3.0	3.0	3.0	3.0
Choline bitartrate	2.5	2.5	2.5	2.5	2.5
tert-butylhydroquinone	0.014	0.014	0.014	0.014	0.014

G1 and G2: AIN-93G diet; G3: AIN-93G diet containing 10% (w/w) of brewers' rice; G4: AIN-93G diet containing 20% (w/w) of brewers' rice; G5: AIN-93G diet containing 40% (w/w) of brewers' rice.

**Table 2:** Serum ALT, ALP, AST, creatinine, and urea in AOM-induced liver and kidney toxicity after twenty-week treatment with brewers' rice.

	ALT (U/L)	ALP (U/L)	AST (U/L)	Creatinine (umol/L)	Urea (mmol/L)
Normal	45.10 ± 6.59^a^	87.00 ± 22.17^a^	141.60 ± 23.23^a^	73.00 ± 14.02^a^	6.80 ± 1.69^a^
AOM only	68.80 ± 10.07^b^	164.00 ± 52.57^b^	201.90 ± 75.56^a^	85.00 ± 10.64^a^	7.00 ± 0.48^a^
AOM + 10% (w/w) of brewers' rice	62.60 ± 9.04^b^	109.00 ± 31.13^ab^	159.20 ± 20.89^a^	78.00 ± 11.76^a^	6.70 ± 1.19^a^
AOM + 20% (w/w) of brewers' rice	56.30 ± 10.69^ab^	134.00 ± 55.86^ab^	187.10 ± 34.70^a^	78.00 ± 10.16^a^	7.80 ± 1.62^a^
AOM + 40% (w/w) of brewers' rice	55.80 ± 11.02^ab^	94.00 ± 20.77^a^	179.70 ± 44.99^a^	76.00 ± 6.87^a^	6.70 ± 0.97^a^

ALT: alanine transaminase; ALP: alkaline phosphatase; AST: aspartate transaminase.

Values are expressed as mean ± SD (*n* = 6). Values in the same column with different superscript letter indicate significant difference by Tukey's test (*P* < 0.05).

**Table 3:** Scores of liver damage after treatment with brewers' rice in AOM-induced rats.

	After 8 weeks of treatment	After 20 weeks of treatment
Normal	0.80 ± 0.45^a^	1.20 ± 0.45^a^
AOM only	2.60 ± 1.52^a^	4.60 ± 0.55^b^
AOM + 10% (w/w) of brewers' rice	2.20 ± 1.64^a^	4.20 ± 0.45^bc^
AOM + 20% (w/w) of brewers' rice	2.00 ± 1.00^a^	3.40 ± 1.34^cd^
AOM + 40% (w/w) of brewers' rice	1.60 ± 0.55^a^	2.80 ± 1.30^d^

Each value is expressed as mean ± SD (*n* = 6). Values in the same column with different superscript letter indicate significant difference by Tukey's test (*P* < 0.05).

**Table 4:** Scores of kidney damage after treatment with brewers' rice in AOM-induced rats.

Groups	After 8 weeks of treatment				
	Normal	AOM only	AOM + 10% (w/w) of brewers' rice	AOM + 20% (w/w) of brewers' rice	AOM + 40% (w/w) of brewers' rice
Vacuolation and the degeneration of the tubules	0	0	0	0	0
Tubular dilatation and deposition of protein cast within the tubular lumina of the kidney	0	0	0	0	0
Degree of inflammation	0^b^	0.67 ± 0.52^a^	0^b^	0^b^	0^b^
Degree of necrosis	0	0	0	0	0

Each value is expressed as mean ± SD (*n* = 6). Value in the same row with different superscript letter indicates significant difference by Tukey's test (*P* < 0.05).

**Table 5:** Scores of kidney damage after treatment with brewers' rice in AOM-induced rats.

Groups	After 20 weeks of treatment				
	Normal	AOM only	AOM + 10% (w/w) of brewers' rice	AOM + 20% (w/w) of brewers' rice	AOM + 40% (w/w) of brewers' rice
Vacuolation and the degeneration of the tubules	0	0	0	0	0
Tubular dilatation and deposition of protein cast within the tubular lumina of the kidney	0	0	0	0	0
Degree of inflammation	0^a^	0.83 ± 0.41^b^	0.67 ± 0.52^b^	0^a^	0^a^
Degree of necrosis	0	0	0	0	0

Each value is expressed as mean ± SD (*n* = 6). Value in the same row with different superscript letter indicates significant difference by Tukey's test (*P* < 0.05).
